# Molecular Detection of Lymph Node Metastases in Lung Cancer Patients Using the One-Step Nucleic Acid Amplification Method:Clinical Significance and Prognostic Value

**DOI:** 10.3390/cells11244010

**Published:** 2022-12-12

**Authors:** María Teresa Hermida-Romero, Lara S. Estévez-Pérez, Begoña O. Alen, Florencia Picchi, Ricardo Fernández-Prado, Mercedes de la Torre-Bravos, Ángel Concha

**Affiliations:** 1 Department of Anatomical Pathology, University Hospital Complex A Coruña, As Xubias 84, 15006 A Coruña, Spain; 2 Molecular Biology Area, Department of Anatomical Pathology, INIBIC, University Hospital Complex A Coruña, 15006 A Coruña, Spain; 3 Fundación Profesor Novoa Santos External R&D, 15006 A Coruña, Spain; 4 Department of Thoracic Surgery, University Hospital Complex A Coruña, 15006 A Coruña, Spain; 5 Biobank of A Coruña, INIBIC, 15006 A Coruña, Spain

**Keywords:** lung cancer, metastasis, OSNA assay, lymph nodes, cytokeratin 19

## Abstract

The one-step nucleic acid amplification (OSNA) method allows for the quantitative evaluation of the tumor burden in resected lymph nodes (LNs) in patients with lung cancer. This technique enables to detect macro and micrometastases, facilitating the correct classification of patients for appropriate follow-up of the disease after surgery. Of 160 patients with resectable lung cancer whose LNs were examined by OSNA, H&E and CK19 IHC between July 2015 and December 2018, 110 patients with clinical stages from IA1 to IIIB were selected for follow-up. LN staging in lung cancer by pathological study led to understaging in 13.64% of the cases studied. OSNA allowed to quantify the tumor burden and establish a prognostic value. Patients with a total tumor load of ≥1650 cCP/uL were associated with a significantly increased likelihood of recurrence. Moreover, the survival of patients with <4405 cCP/uL was significantly higher than patients with ≥4405 cCP/uL. The OSNA assay is a rapid and accurate technique for quantifying the tumor burden in the LNs of lung cancer patients and OSNA quantitative data could allow to establish prognostic values for recurrence-free survival and overall survival in this type of malignancy.

## 1. Introduction

Lymph node metastasis (LNM) detection at the time of diagnosis is considered a predictor of survival in lung cancer [1,2]. The one-step nucleic acid amplification (OSNA) method quantifies cytokeratin 19 (CK19) mRNA expression, and is used in clinical practice for the diagnosis of LNM in several types of tumors, such as breast [3], gastric [4] or colorectal cancer [5], and recently in prostate [6] and gynaecological malignancies [7]. Its application in head and neck/thyroid is also being evaluated [8,9]. However, only a small number of studies have been reported in relation to bronchopulmonary malignancies [10,11,12]. In lung cancer, histopathological examination was considered the gold standard method for disease staging, but the significant likelihood of false negatives with this technique can lead to an incorrect diagnosis, with repercussions for patient management and for overall survival (OS). The literature reflects up to 24.1% of false negatives in haematoxylin and eosin (H&E) staging [13], mainly due to the presence of micrometastases or isolated tumor cells (ITCs) not having been identified. The failure to detect these microdeposits by histopathological methods results in an underestimation of the stage and the understanding of the disease. This causes a recurrence rate of up to 30% following surgical treatment [14], which is closely related to OS. Patients with micrometastatic foci that were not diagnosed had significantly shorter recurrence-free survival (RFS) and OS compared to N0, but significantly longer than those with N1 macrometastases [15]. OSNA analysis is a method that allows the quantitative evaluation of tumor volume and the detection of these micrometastases, which would facilitate the correct classification of patients for appropriate follow-up of the disease after surgery. 

This work is part of a previous study that demonstrated the reliability, validity and accuracy of OSNA in the detection of mediastinal LNM in patients with lung cancer, compared to classical anatomopathological methods (H&E and immunohistochemistry (IHC) of CK19) [10]. The aim of this study was to analyse, through follow-up of the patient cohort, the relationship between LNM detected using both OSNA and anatomo-pathological methods, and the progress of the disease (median follow-up = 42.8 months; interquartile range (IQR) 33.72–55.36 months), as well as to establish a cut-off level related to recurrence and survival in a group of 110 patients. 

## 2. Materials and Methods

### 2.1. Patients and Human Samples

**Scheme 1:**
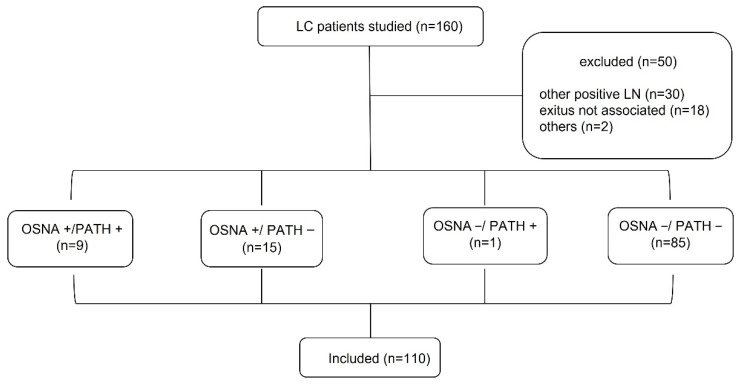
Baseline characteristics of the patients included in the follow-up study.

Patients with resectable lung cancer whose LNs were studied by OSNA, H&E and CK19 IHC between July 2015 and December 2018. Of the 160 patients belonging to the study, 110 subjects with clinical stages from IA1 to IIIB according to the latest AJCC Cancer Staging Manual of the TNM classification for lung cancer [16] were chosen for follow-up. Patients with lung cancer and positive LNs not studied by OSNA, as well as those who died from events not associated with lung cancer, were excluded. The flowchart is shown in Scheme 1. Follow-up data were collected from medical records and the median follow-up was 42.8 (IQR 33.72–55.36) months. The detailed characteristics of the group are provided in Table 1.

The diagnosis and treatment of the patients were conducted by the University Hospital Complex of A Coruña (CHUAC) multidisciplinary committee on thoracic tumors, which bases their decisions on international scientific society guidelines in order to harmonise practices. All patients underwent an endobronchial ultrasound (EBUS) biopsy for pre-surgical staging, and no positive tumor cells were found in any of them.

This study was carried out in accordance with the Declaration of Helsinki. Approval from the Clinical Research Ethics Committee, patients written informed consents custody and sample storage was managed by the Biobank of A Coruña.

### 2.2. Lymph Nodes

A total of 486 LNs from 110 patients were studied by OSNA, H&E and CK19 IHC. The mean value was 4.42 LNs/patient (ranging from 1 to 11 LNs/patient). Only one half of the lymph node was assessed using the OSNA method, and the removal and preparation of LNs for histological examination, CK19 IHC and OSNA (Sysmex, Kobe, Japan), was performed as previously described [10]. Histopathological results were analyzed by two independent pathologists. There were no cases of non-consensus, so it was not necessary to use an additional technique or refer to a third expert pathologist. The results of the molecular assay were expressed as CK19 mRNA copy numbers per microlitre (mRNA CK19 cCP/uL). The cut-off level for micrometastasis was set at 250—4999 cCP/uL and over 4999 cCP/uL was considered macrometastasis; less than 250 cCP/uL was considered negative. These values are those established for analysis of the whole lymph node [17], not for half of the node, as was our case. The details of the LNs included are provided in Table 2. 

Patients were classified according to molecular and histological findings [18]. All results obtained by anatomopathological techniques were concordant (H&E staining method and CK19 IHC) and these results are indicated with PATH. Four distinct groups were defined: OSNA+/PATH+; OSNA+/PATH−; OSNA−/PATH+ and OSNA−/PATH−. The complete characteristics of all patients and associated lymph nodes are described in Table S1.

### 2.3. Study Definitions

Total tumor load (TTL) was defined as the sum of the total mRNA CK19 cCP/uL detected by OSNA in all positive LNs per patient. Lymph node metastases (LNMs) were considered positive lymph nodes by OSNA, H&E-CK19 IHC or both. Recurrence-free survival (RFS) was defined as the period with no evidence of disease. 

### 2.4. Statistical Analysis

Differences between the CK19 mRNA levels obtained by OSNA for PATH+ and PATH− nodes were calculated using the non-parametric Mann-Whitney U test. Descriptive statistics were used for quantitative mean (or median) and standard deviation (SD) or IQR values. The Kaplan-Meier model was used for the calculation of OS and RFS. Recurrence and death events were studied using ROC analysis and the Kaplan-Meier model. The median survival can be obtained from the Kaplan-Meier curve as the time in which the curve changes from a probability of survival greater than 0.5 to less than 0.5. ROC analysis was performed to evaluate the performance of the two techniques and to establish cut-off levels. Statistical analyses were calculated using IBM SPSS^®^ Statistics v27 and R statistic software v4.0.2. The statistical significance was determined at α-limit = 5%.

## 3. Results

### 3.1. Concordance Rate between OSNA and Histopathological Methods (PATH)

**Figure 1:**
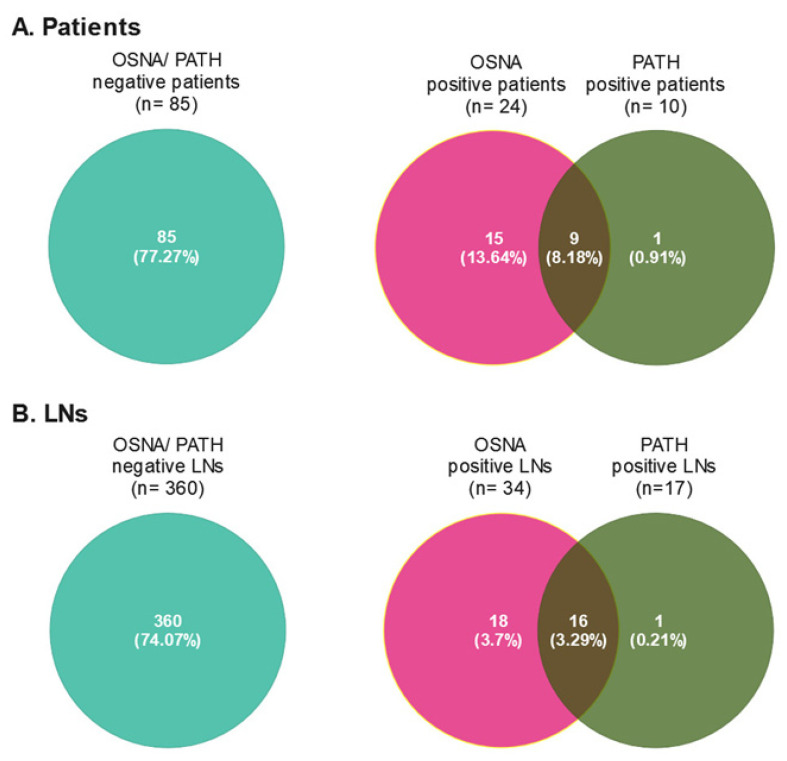
Venn diagrams comparing the different techniques (OSNA and PATH (H&E and IHC)) for detection of metastases in patients studied (**A**) and in their individual lymph nodes (**B**). The percentage is based on the total number of patients and LNs included in the study.

After evaluating the LN results of all patients included in the follow-up (1–11 LNs per individual were evaluated), concordance was established in 85.45% (94/110 patients; Figure 1A). Eighty-five patients were OSNA−/PATH−. 8.18% (9/110) were OSNA+/PATH+. All positive patients showed molecular and histological macrometastasis except for one patient (ID80;0.91%), (1/110), with 310 cCP/uL by the OSNA method and histological macrometastatic foci. However, one patient (ID16;0.91%) (1/110) was found to be negative by histopathological examination, and positive by OSNA with 170720 cCP/uL (macrometastasis), most likely due to tumor allocation bias. Finally, in just one case (ID94;0.91%) (1/110), the histopathological examination was positive, while the OSNA method detected less than 250 cCp/uL, also most likely due to tumor allocation bias, rather than a sensitive method issue. Despite being defined as a pN2 patient by anatomopathological methods, no recurrence or death had occurred at the end of the follow-up period. False negative cases would therefore be exceptional if whole LNs were analyzed by OSNA. In relation to the total number of LNs (Figure 1B), 486 individual LNs were studied. 3.29% of the total (16/486) were OSNA+/PATH+; 18 LNs (3.70%; 18/486) were OSNA+/PATH−; 1 LN (0.21%;1/486) was OSNA−/PATH+; and finally, 92.80% (451/486) were negative in molecular and histological determinations. Moreover, 29.06% of LNs analysed with the OSNA technique performed as sentinel lymph node function (34/117; Table 2). 

Regarding the disease stage, changes in the pTNM are detailed in Table 3. 90.91% of patients (100/110) were LN negative (pN0) by histological analysis. Fifteen of these pN0 patients were OSNA positive (mN1-2), resulting in an upstaging of 13.64% of all patients analysed by molecular technique. This restaging would affect 60% of patients with LNM [positive patients i.e., OSNA+ and/or PATH+ (15/25)]. In all, 20% of this cohort would change from N0 to N1 (3/15), and 80% from N0 to N2 (12/15) (see Table 3). Therefore, microscopic examination led to an understaging of 13.64% of the 110 patients included in the study. No restaging was observed in OSNA+/PATH+ cases. Finally, OSNA−/PATH+ was actually a histological N2 (Table 3).

### 3.2. Study of the Sample Distribution According to TTL of mRNA CK19 cCP/uL in the OSNA+/PATH+ and OSNA+/PATH− Cohorts

**Figure 2:**
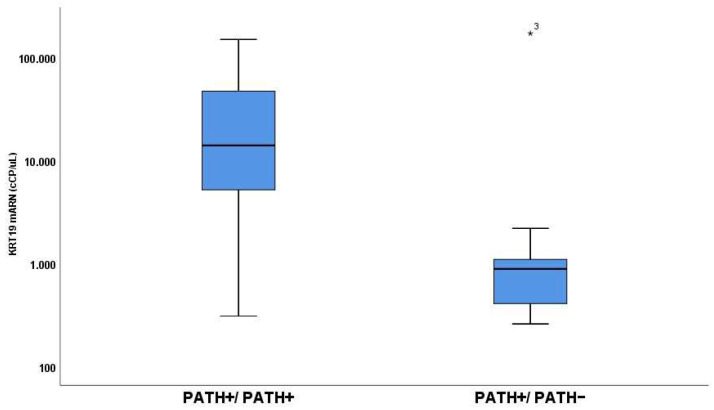
Box plot showing the distributions of the two OSNA+/PATH+/− expression profiles. The y-axis indicates the TTL of mRNA CK19 cCP/uL in patients on a logarithmic scale. Asterisk (*) denotes a data of patient 16 with a very high load of copies of mRNA CK19.

The TTL of mRNA CK19 cCP/uL in patients was calculated individually based on their profile results from both tests (OSNA+/PATH+/−). A box plot diagram was created to visualise the distributions of both profiles, as shown in Figure 2. The median TTL of mRNA CK19 cCP/uL for OSNA+/PATH+ patients was 14,000 cCP/uL (IQR 5100–82,500 cCP/uL), and for OSNA+/PATH− cases it was 890 cCP/uL (IQR 270–2200 cCP/uL). On the other hand, the mean ± SD yielded a value for OSNA+/PATH+ = 40,657.78 ± 54,876.98 cCP/uL vs. OSNA+/PATH− = 19,696.67 ± 56,636.96 cCP/uL. Interestingly, in this profile, we found a patient with 170,720 cCP/uL (Figure 2). Despite this, both groups were found to be significantly different, with a non-parametric Mann-Whitney U test yielding a *p*-value of 0.0043.

### 3.3. Concordance Overall Survival of the Patients Studied by OSNA. Comparison and Prognostic Significance 

**Figure 3:**
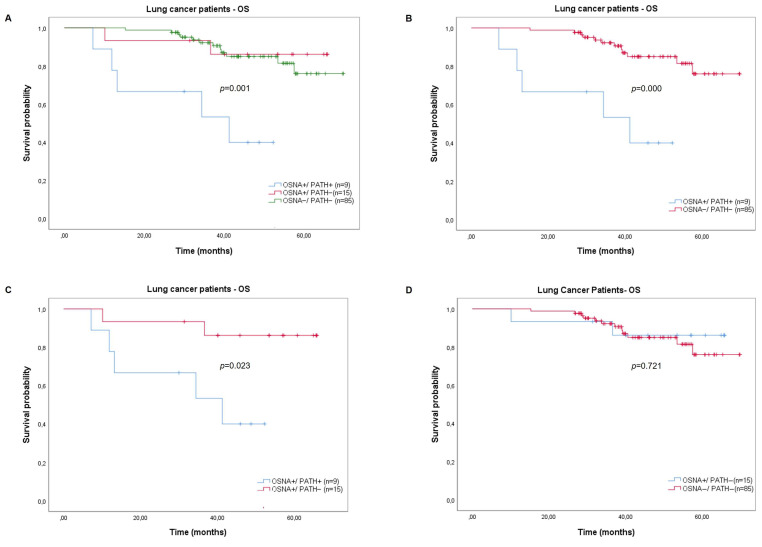
Kaplan-Meier curves comparing OS distribution according to three patient profiles OSNA+/PATH+, OSNA+/PATH− and OSNA−/PATH− (**A**). OS of lung cancer patient OSNA+/PATH+ vs. OSNA−/PATH− (**B**); OSNA+/PATH+ vs. OSNA+/PATH− (**C**); and OSNA+/PATH− vs. OSNA−/PATH− (**D**).

The Kaplan-Meier model representing the OSNA+/PATH+, OSNA+/PATH− and OSNA−/PATH− profiles were used to evaluate OS (Figure 3A). The fourth one, OSNA−/PATH+ (*n* = 1), was not statistically significant and therefore was not represented. The model yielded a *p*-value = 0.001, so we found significant differences in OS between the different profiles. To establish these differences, we ran Kaplan-Meier models between groups 2 to 2. As seen above, each of these profiles was characterised by a particular TTL of mRNA CK19 cCP/uL distribution. In patients with concordant positive results (OSNA+/PATH+), the median number of months of survival time was 34.37 (IQR 12.54–47.33 months), an unfavourable OS as opposed to patients with discordant results (OSNA+/PATH−), whose median number of months of survival time was 53.43 (IQR 40.07–64.93 months), (*p*-value = 0.023; Figure 3C). 

In the same way and, as expected, we observed significant differences in OS (*p*-value = 0.000) between OSNA+/PATH+ patients (median number of months of survival time = 34.37; IQR 12.54–47.33 months) vs. OSNA−/PATH− (median number of months of survival time = 41.87; IQR 33.70–55.09 months; Figure 3B).

Finally, there were no significant differences (*p*-value = 0.721, Figure 3D) between OSNA+/PATH− (≥250 cCP/uL, median 890 cCP/uL; IQR 270–2200 cCP/uL) and OSNA−/PATH− (<250 cCP/uL) profiles. The median TTL of CK19 cCP/uL of the OSNA+/PATH− patients was not elevated enough to differ from non-LNM patients (Figure 2). Interestingly, median survival time was also longer (53.43 months) than in the case of the OSNA−/PATH− group (41.87 months). However, these differences were not significant. The small number of patients in the OSNA+/PATH− group (*n* = 15) vs. the OSNA−/PATH− (*n* = 85) group could be a limitation in predicting survival time. A larger cohort of cases would be necessary for profiling OS more accurately and to confirm that the presence of molecular micrometastasis could be critical for survival in this type of malignancy. 

We also performed a Cox regression model to explore possible demographic (sex, age), clinical (stage), or histological (histological type, subtypes…) confounding variables that could be influencing the model. We have not found any significance between them and CK19 levels (Supplementary Material Table S2). We also made the minimum required clinical model (sex/age), and we observed that it was not significant either and that it also distorts the ability of CK19 to predict OS and DFS in lung cancer patients (Supplementary Material Table S3 and Figure S1).

### 3.4. A Prognostic Cut-Off Value Can Be Determined by OSNA for Recurrence-Free (RFS) and Overall Survival (OS) in Lung Cancer Patients

**Figure 4:**
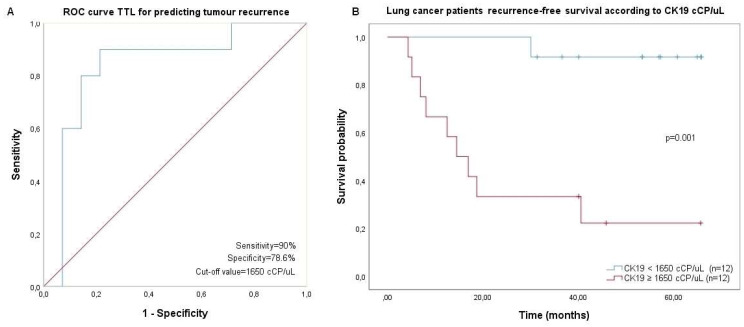
ROC curve for recurrence. Area = 0.836 95% IC (0.659–1.000), *p*-value = 0.006 (**A**). OS curve for patients without recurrence (<1650 cCP/uL) vs. patients with recurrence, *p*-value = 0.001 (**B**).

**Figure 5:**
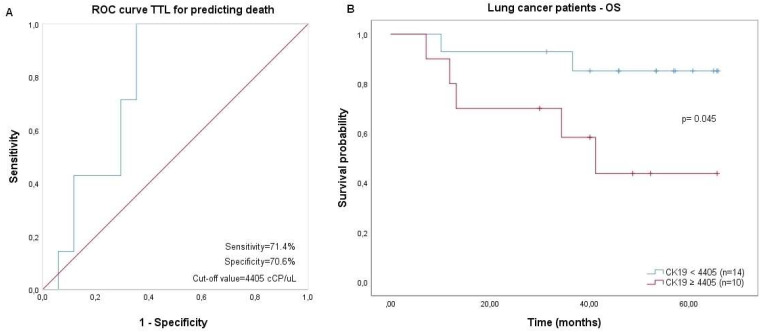
ROC curve for predicting death. AUC = 0.773 95% IC (0.588–0.959), *p*-value = 0.039 (**A**). OS curve for patients with high survival probability (<4405 cCP/uL) vs. patients with low survival probability (≥4405 cCP/uL) *p*-value = 0.045 (**B**).

In order to establish a prognostic value for recurrence and survival using the OSNA method in bronchopulmonary malignancies, a ROC curve analysis was performed (Figure 4 and Figure 5).

The analysis of half of the node yielded a cut-off value of 1650 cCP/uL, with a sensitivity of 90.0% and a specificity of 78.6% for predicting tumour recurrence (Figure 4); and a cut-off point of 4405 cCP/uL (71.4% sensitivity and 70.6% specificity) for predicting survival (Figure 5). With this data, when the cut-off value was set at <1650 cCP/uL vs. ≥1650 cCP/uL, we obtained a significantly different RFS between patients (*p*-value = 0.001). A TTL of ≥1650 cCP/uL was significantly associated with an increased probability of recurrence. Moreover, and in line with the above, we also observed that survival among patients that showed <4405 cCP/uL was significantly higher compared to those with a number of detected copies of ≥4405 cCP/uL (*p*-value = 0.045; Figure 5). 

**Figure 6:**
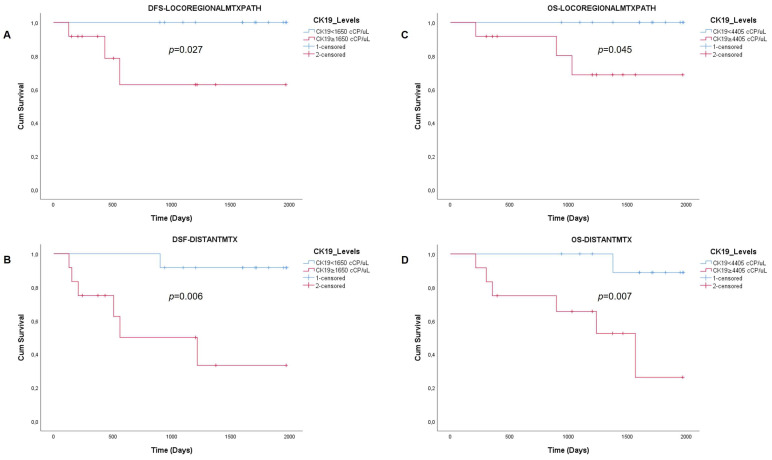
Kaplan-Meier curves comparing OS and DFS distribution in patients with high or low levels of CK19, and the appearance of loco-regional and distant metastasis. (**A**). DFS curve for patients harboring loco-regional metastasis with high survival probability (<1650 cCP/uL) vs. patients with low survival probability (≥1650 cCP/uL) *p*-value = 0.006. (**B**) DFS curve for patients harboring distant metastasis with high survival probability (<1650 cCP/uL) vs. patients with low survival probability (≥1650 cCP/uL) *p*-value = 0.045. (**C**) OS curve for patients harboring loco-regional metastasis with high survival probability (<4405 cCP/uL) vs. patients with low survival probability (≥4405 cCP/uL) *p*-value = 0.045. (**D**) OS curve for patients harboring distant metastasis with high survival probability (<4405 cCP/uL) vs. patients with low survival probability (≥4405 cCP/uL) *p*-value = 0.006.

Finally, we used The Kaplan-Meier model to observe differences in OS and DFS in patients with cumulative local or distant metastases. As shown in Figure 6, we observed significant differences in both OS and DFS in patients with loco-regional metastases and in those who developed distant metastases, depending on CK19 levels detected by OSNA assay.

Therefore, for the first time, we are describing a cut-off value for recurrence in lung cancer of 1650 cCP/uL in the molecular study of LNs analysed by OSNA. This TTL, in half a lymph node, provides a prognostic value for this type of tumor, allowing patients to be classified into different risk groups according to RFS. Furthermore, a cut-off value of 4405 cCP/uL could be established to predict survival rates in this type of patient.

## 4. Discussion

The early detection of bronchopulmonary cancer is a key factor for improving the possibilities of finding a successful treatment and increasing patient survival rates. However, despite medical efforts, the five-year survival rate for lung cancer is still too low [19]. OSNA is a validated diagnostic method in breast, colon, gastrointestinal, prostate, lung and gynaecological malignancies [3,4,5,6,7,8,9]. The application of the OSNA technique for intraoperative analysis of sentinel lymph nodes in breast, colon and gastric cancer has been an alternative for several years [4,20,21], and various articles have shown its potential usefulness for colon cancer staging [22,23,24]. This work is a patient follow-up study on lung cancer related to previous research conducted between July 2015 and December 2018 [10]. The prognostic value of OSNA was studied by an approximation of TTL. This value was determined from half of the positive lymph nodes examined by OSNA. The results yielded high values for accuracy in the detection of LNMs and concluded that the OSNA assay might be a useful and sensitive method for diagnosing LN metastases in lung cancer. 

However, there are still many issues to be established. Could OSNA be useful for intraoperative decision-making or for sentinel lymph node detection? Could CK19 mRNA levels be more informative for patient diagnosis and even prognosis? The lack of long-term and conclusive studies on lung cancer means that more exhaustive studies are required in order to answer such questions. 

Regarding the first matter, sentinel lymph node biopsy is a technique that revolutionised the surgical management of cutaneous melanoma and breast cancer. In recent years, thoracic surgeons have been working to apply this technique to lung cancer, but it has not had the same impact as in other malignancies [1,25]. In this study, 29.06% of the analysed LNs were defined as sentinel LNs, but its relevance is yet to be determined. Interinstitutional variability and the lack of multicentre prospective data make clinical adoption of the technique difficult, and its applicability is still being discussed. In our study, the lymphatic resection areas most susceptible to being drained by the tumor were selected, so we used an adapted sentinel lymph node-like system. Our subsequent results supported the idea that, in the near future, the OSNA method could be a feasible and suitable technique for sentinel lymph node detection in bronchopulmonary malignancies. 

The accuracy of molecular methods for staging in lung cancer has already been demonstrated, but it may also be useful for patient prognosis. We reported an RFS cut-off level for lung cancer for the first time. The quantification of the tumor burden for the molecular staging of LNs could become an objective tool for risk stratification in this type of patient, as in the case of colorectal cancer. Rakislova et al. reported a median TTL value from OSNA positive cases of 1940 cCP/uL in the individual cohort, and pN0 patients with positive OSNA results yielded a median TTL of 1540 cCP/uL (IQR 620–3600 cCP/uL) [23]. An identical TTL amount of 1550 cCP/uL in pN0 patients was established previously through other work [22]. Recently, Archilla et al. also showed that a TTL of ≥6000 cCP/uL in colorectal cancer was associated with worse OS and disease-free survival rates [26]. However, the metrics in breast cancer may diverge from this. Vieites et al. established a predictive cut-off point > 25,000 mRNA cCP/uL associated with a higher risk of disease recurrence in breast cancer patients [27]. Our results showed that the median TTL of mRNA CK19 cCP/uL for positive patients was 14,000 cCP/uL (IQR 5100–82,500 cCP/uL), and for discordant cases, it was 890 cCP/uL (IQR 270–2200 cCP/uL). We did not find significant differences between the former group and the negative (<250 cCP/uL) profile, however, a cut-off value of 1650 cCP/uL with a sensitivity of 90.0% and a specificity of 78.6% for predicting tumor recurrence, and a cut-off point of 4405 cCP/uL (71.4% sensitivity and 70.6% specificity) for predicting survival could be established for the first time in NSCLC. These differences are also significant when analyzing patients with loco-regional and distant metastases. This data could be crucial for decision-making and patient management in this type of pathology. The procedures and treatments recommended for lung cancer patients depend not only on the histopathological diagnosis and molecular profile of the tumor, but also on the clinical stage of the disease. The implementation of OSNA in clinical practice could also have a significant impact on staging. In this study, we saw that 13.64% of 110 patients were susceptible to restaging by the molecular method. Similar data was found in the literature. In colorectal cancer, Yamamoto et al. reported a rate of 11.3% of understaged cases, and similar rates were found in other malignancies [28]. This could be an important consideration for the clinical management of disease. The other main question in this regard is if OSNA positive lung cancer patients should receive adjuvant chemotherapy after surgery. This is closely related to the possibility of small niches of tumor cells remaining after surgical procedures. In this study, all OSNA+/PATH− patients were susceptible to restaging by the molecular method, from N0 to N1 (20.00%) or N2 (80.00%). In recent years, meta-analyses have been conducted on this subject, and it has been observed that administering chemotherapy after surgery had a beneficial effect on lung cancer patients [29]. In this context, the OSNA assay could provide additional information for pathology management. 

In the past decade, there has been controversy over the prognostic significance of ITCs and micrometastasis in different tumors. In breast cancer, ITCs and micrometastases do not appear to interfere with OS [30,31]. In patients with colorectal cancer, disease recurrence was significantly increased with the presence of micrometastases in comparison with absent occult tumor cells. In contrast, disease recurrence was not increased in the presence of ITCs [32]. On the other hand, in regard to lung cancer, the controversy continues and is still unresolved [33,34,35]. The results obtained in this work highlight the importance of detecting micrometastases in lung cancer. In this regard, the accuracy of OSNA could estimate a micrometastatic prognostic value.

Moreover, previous studies have shown that the association between OSNA positivity and disease progression is low (with a cut-off level of ≥250 mRNA CK19 cCP/uL, according to technical specifications). Recent studies have proposed increasing this cut-off point to 615 in order to enhance the association between both methods. This variation would mean that a higher cut-off level could be more relevant in the case of bronchopulmonary tumors [14]. In our study, as described above, the Kaplan-Meier curve showed that patients with discordant results, OSNA+/PATH− profile, did not differ significantly in OS compared to OSNA−/PATH− patients. However, in four of these 15 total patients, a CK19 mRNA level of ≥1650 cCP/uL was detected. This data implies that 26.6% of cases exceeding the cut-off recurrence value were not detected. In addition, TTL would allow patients to be classified into risk groups for disease recurrence and survival probability by defining pN more specifically. Although this study only analysed half of the lymph node using the OSNA technique, the TTL with prognostic value was in the range of a micrometastatic rate of <5000 cCP/uL. The TTL value also defines the patient as a whole, but the distribution of the tumor load will vary depending on each individual. There will be patients that exceed the cut-off point with only one LN, and others who will exceed it with two or more affected nodes. In breast cancer, it has been shown that the presence of a single macrometastasis might have an intermediate prognostic value, while two macrometastases or more were associated with a poorer prognosis [36]. Furthermore, with melanoma, the size of the largest diameter of the lymphatic metastasis is important for characterising the tumor and is correlated to survival [37,38]. Different criteria were followed depending on the type of tumor. However, TTL could become a disruptive value in lung cancer. Would the number of affected LNs in these types of malignancies be indifferent, or would the prognosis change if the tumor load was distributed differently? This point must be established, as the current staging of lung cancer considers the location of the LNs, but not the number affected [16]. 

## 5. Conclusions

In conclusion, our results showed for the first time that TTL by the OSNA method has a predictive value in lung cancer. Therefore, if only histopathological procedures are to be the basis of TNM, then staging must be discussed. Multicentre prospective studies with a larger sample size will be essential for establishing a robust cut-off value that will be crucial for decision-making, both intraoperatively and in the treatment and follow-up period of bronchopulmonary malignancy patients. 

## Figures and Tables

**Table 1:** Baseline characteristics of the patients included in the follow-up study.

Variables	N°	%	Variables	N°	%	Variables	N°	%
Patients	110		Pathological stage	110		Histology	110	
Male	73	66.36	IA1	6	5.45	Adenocarcinoma	65	59.09
Female	37	33.64	IA2	21	19.09	Squamous cell carcinoma	25	22.73
Age (yr)			IA3	16	14.55	Adenosquamous carcinoma	3	2.73
Mean (range)	64.73 (22–85)		IB	26	23.64	Carcinoid NET	8	7.27
Surgical type of resection	110		IIA	9	8.18	Large-cell carcinoma	8	7.27
			IIB	17	15.45	Pleomorphic carcinoma	1	0.91
			IIIA	14	12.73			
Lobectomy	103	93.64	IIIB	1	0.91	Molecular N status OSNA	110	
			Pathological N status PATH	110				
Bi-lob	7	6.36	pN0	100	90.91	mN0	86	78.18
			pN1	3	2.73	mN1	5	4.55
			pN2	7	6.36	mN2	19	17.27

**Table 2:** Characteristics of LNs of 110 patients included in the study.

Patient Profile	110 Patients	486 LNs Studied	451 Negative LNs	35 Positive LNs	% Positive LNs
**OSNA+/PATH+**	9	48	32	16	3.29
**OSNA+/PATH−**	15	69	51	18	3.70
**OSNA−/PATH+**	1	9	8	1	0.21
**OSNA−/PATH−**	85	360	360	0	0.00

**Table 3:** Changes in the pTNM staging depending on OSNA and PATH (H&E and IHC) results. Abbreviations: mN: molecular TNM, OSNA: one-step nucleic acid amplification, H&E: haematoxylin-eosin, IHC: immunohistochemistry.

OSNA/PATH	OSNA		PATH		Cases
Profile	mN	Stage	pTNM	Stage	n
**OSNA+/PATH+**	mN1	IIA	T2aN1M0	IIA	1
	mN1	IIB	T2bN1M0	IIB	2
	mN2	IIIA	T2bN2M0	IIIA	5
	mN2	IIIB	T3N2M0	IIIB	1
**OSNA+/PATH** **−**	mN1	IIB	T2bN0M0	IB	2
	mN2	IIIA	T2aN0M0	IB	4
	mN2	IIIA	T1aN0M0	IB	4
	mN1	IIIA	T3N0M0	IIB	1
	mN2	IIIA	T1cN0Mx	IA3	2
	mN2	IIIA	T1bN0M0	IB	2
**OSNA** **−** **/PATH+**	mN0	IB	T2aN2Mx	IIIA	1

## Data Availability

The data presented in this study are available in the article. Any other related-information or document does not present in this study are available on request.

## Supplementary Materials

The following supporting information can be downloaded at: https://www.mdpi.com/article/10.3390/cells11244010/s1, Table S1. Characteristics of the patients and lymph nodes included in the study; Table S2. Cox Regression Model; Table S3. Cox Minimal Clinical Model.
